# Effects of Reinforcement Materials and Mechanical Retention on the Flexural Strength and Deflection of Repaired Denture Base Resin

**DOI:** 10.7759/cureus.97722

**Published:** 2025-11-25

**Authors:** Masatoshi Iwasaki, Takashi Iida, Yuki Ishii, Yuichiro Yamakawa, Mika Honda-Sakaki, Yoshihiro Iwata, Yuna Yanagizono, Chihiro Watanabe-Iwasaki, Osamu Komiyama

**Affiliations:** 1 Department of Prosthodontics and Oral Rehabilitation, Nihon University School of Dentistry at Matsudo, Chiba, JPN; 2 Department of Anatomy, Nihon University School of Dentistry at Matsudo, Chiba, JPN; 3 Department of Pediatric Dentistry, Nihon University School of Dentistry at Matsudo, Chiba, JPN

**Keywords:** denture repair, domiciliary dental care, mean flexural strength, prosthodontics-related dental material, resin reinforcement

## Abstract

The increasing prevalence of denture base fractures among older patients underscores the clinical importance of effective repair strategies. The present study aimed to evaluate the effects of mechanical retention forms and reinforcement materials on the flexural strength and deflection of repaired denture base resin. Rectangular specimens (65 × 10 × 2.5 mm) were prepared from autopolymerizing resin and divided into seven groups: control (C), resin-only (R), primer-treated (RP), two interlocks (IL2), four interlocks (IL4), two parallel wires (W2), S-shaped wire (WS), and metal mesh (MM). Repairs were performed using autopolymerizing resin and tested under three-point bending (span: 50 mm; crosshead speed: 5 mm/min). The C group exhibited a flexural strength of 157.1 ± 14.3 MPa, while the R group showed the lowest (54.4 ± 10.2 MPa). Primer application improved strength to 92.2 ± 19.0 MPa, and wire reinforcement, particularly the W2 configuration, restored flexural strength comparable to the C group (163.5 ± 18.7 MPa). All repair groups showed reduced deflection relative to the C group, with the W2 group demonstrating the greatest preservation of flexibility (4.9 ± 1.0 mm vs. 7.7 ± 1.4 mm). These findings indicate that primer treatment combined with suitable reinforcement effectively enhances the mechanical properties of repaired denture base resin. Although full recovery of original strength and flexibility was not achieved, wire reinforcement provided superior load resistance, while metal mesh offered practical advantages for chairside or domiciliary repairs. Further studies on primer formulations and long-term durability are recommended to refine clinical repair protocols.

## Introduction

In recent years, many developed countries, including Japan, have experienced rapid population aging, and as a result, the demand for prosthetic treatment for missing teeth is expected to increase further [1-4]. For patients with multiple missing teeth or economic or systemic limitations, removable dentures remain an essential prosthetic option. Consequently, not only the fabrication of new dentures, but also the maintenance and repair of existing dentures have become increasingly important [5-8].

Older patients often continue using the same dentures for extended periods, which increases the risk of denture base fracture as a result of material aging of denture base resin and repeated occlusal loading [5,9-11]. In many clinical cases, such fractures go unnoticed and patients continue using damaged dentures [9-11].

Although remaking the denture is ideal when fracture occurs, many older or homebound patients prefer to continue using their familiar dentures, making repair unavoidable in many cases [5,9-11]. This is particularly common in domiciliary dental care. Fractured dentures, whether complete or partial, remain among the most frequent complications, and improving the prognosis of denture repair is therefore of considerable clinical and social significance, as it directly contributes to maintaining patients’ quality of life [6,9,11-15].

Numerous studies have attempted to enhance the strength of repaired denture base resin by incorporating mechanical retention forms (e.g., interlocking or bevels) or reinforcement materials (e.g., metal wires or meshes) [9-11,16,17]. However, specimen thickness and configurations vary between studies, and few have evaluated specimens reflecting the clinically relevant denture base thickness of approximately 2.5 mm. Moreover, comparative studies examining multiple reinforcement techniques under identical conditions remain limited [11,17,18].

Therefore, in the present study, multiple repair conditions were prepared and compared using a three-point bending test to evaluate flexural strength and deflection. By comparing multiple groups under standardized conditions, this study aimed to clarify the effectiveness of different reinforcement strategies and provide evidence to guide clinical decision-making in denture repair, with particular relevance to chairside and home-visit repair procedures.

The primary outcome of this study was the flexural strength of the repaired denture base resin, while the secondary outcomes included the maximum deflection and the practical handling of the repair procedures.

## Materials and methods

Specimen preparation and reinforcement conditions

An acrylic master plate (66 × 11 × 3 mm) was fabricated and duplicated using an addition-type silicone impression material (Exahiflex Tray Type; GC, Tokyo, Japan). The mold was invested in a dental flask (Whip Mix Co., Louisville, KY, USA) using hard dental stone (New Plastone II; GC) and allowed to set. After setting, autopolymerizing resin (Procast DSP; GC) was poured into the mold and polymerized under pressure using a hydraulic flask press (Dental Press; Morita, Tokyo, Japan) at 0.2 MPa and 55 °C for 30 minutes. After polymerization, specimens were deflasked, excess material was removed, and the surfaces were finished with waterproof abrasive paper up to #800 to achieve final dimensions of 65 × 10 × 2.5 mm.

**Figure 1:**
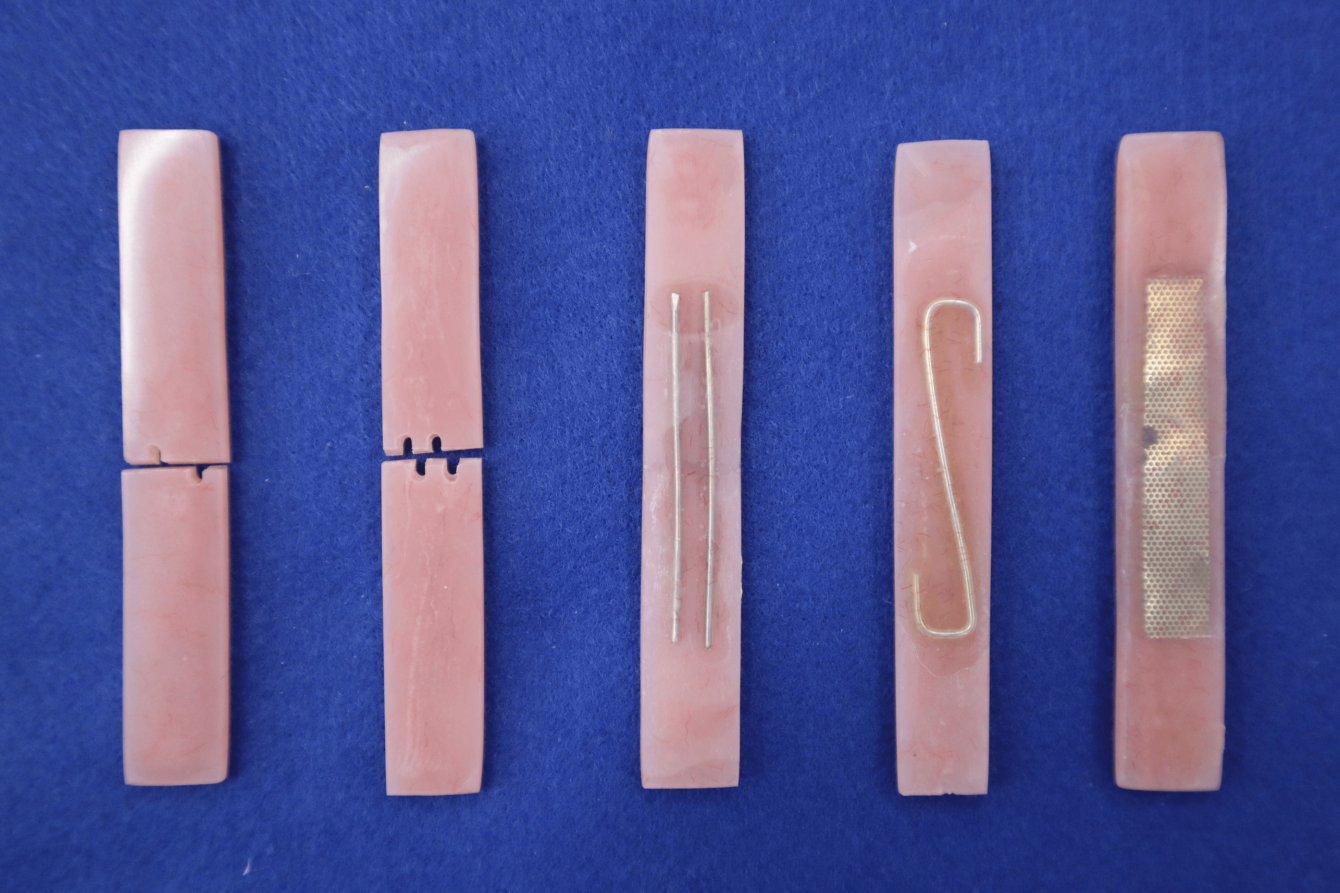
Mechanical retention features of each specimen and representative configurations of reinforcement materials

Table 1 summarizes each specimen group and its corresponding repair conditions. All specimens except the control (C) group were sectioned at the center using a diamond disc. Repairs were performed using autopolymerizing resin (Unifast III, Live Pink; GC). The resin-only (R) group was repaired without any surface treatment. The primer-treated (RP) group received resin primer (Resin Primer; GC) application on the cut surfaces prior to repair. For the interlock (IL) groups, mechanical retention features were created by forming 1 mm-wide, 2 mm-deep grooves on the cut surfaces, ensuring that the grooves did not overlap during repair (Figure 1). For the 10 mm-wide specimens, no grooves were created within 2 mm of either edge. Grooves were applied only to the remaining 6 mm-wide central area, within which specimens with either two or four grooves were prepared.

**Table 1:** Overview of specimen groups

Group name	Abbreviation	Repair details
Control group	C	Control group without repair
Resin-only repair group	R	Repaired with autopolymerizing resin only
Repair with primer group	RP	Repaired after primer treatment
Interlock-2 group	IL2	Repaired after providing mechanical interlock at two sites
Interlock-4 group	IL4	Repaired after providing mechanical interlock at four sites
Two-wire group	W2	Repaired after placement of two 1‑mm‑diameter wires
S-shaped wire group	WS	Repaired after placement of a single 1‑mm‑diameter S‑shaped wire
Metal Mesh group	MM	Repaired after placement of metal mesh

**Figure 2:**
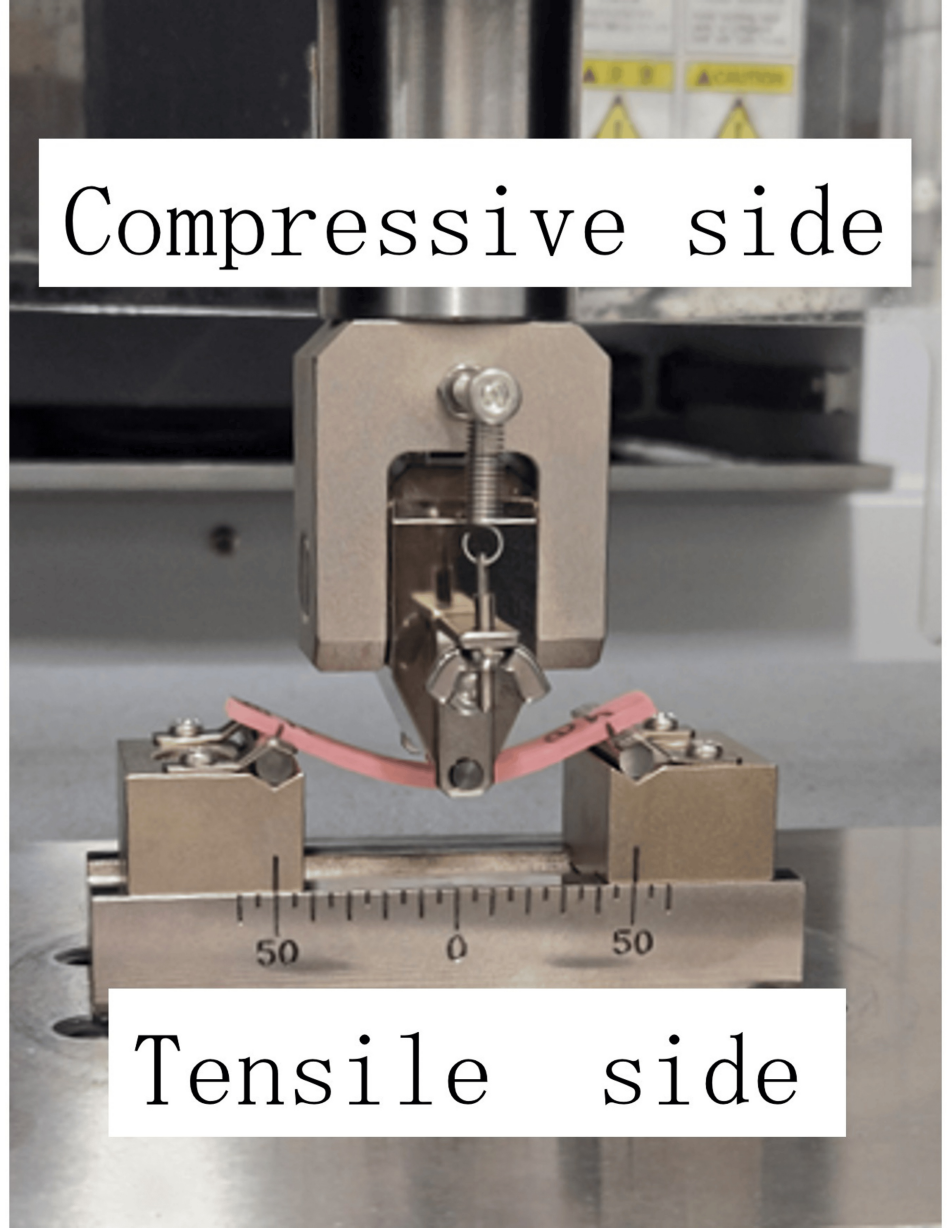
Stress distribution of a specimen during three-point bending

Reinforced groups, including the two-wire (W2) group, S-shaped wire (WS) group, and metal mesh (MM) group, were reinforced on the tensile side using 1-mm Co-Cr wire (Dentsply Sirona, Tokyo, Japan) or 0.25-mm metal mesh (Bonding Base; Tomy Co., Tokyo, Japan), as shown in Figures 1, 2 [9,17-19]. Reinforcements spanned the repair site over a length of 35 mm and a width of 6 mm. The sample size of 16 specimens per group was determined with reference to previous in-vitro studies evaluating the flexural strength of repaired denture base resin. Prior denture repair studies have typically used 8 to 20 specimens per group for three-point bending tests, indicating that the present sample size lies within the commonly accepted methodological range [20,21].

Three-point bending test

All specimens were stored in distilled water at 37 °C for 24 hours prior to testing. Flexural strength and maximum deflection at fracture were measured using a universal testing machine (AGX-100kNV2; Shimadzu, Kyoto, Japan) with a 50-mm span and crosshead speed of 5 mm/min, applying the load at the repair site.

Statistical analysis

Data regarding flexural strength and maximum deflection were analyzed using IBM SPSS Statistics for Windows, Version 23 (Released 2015; IBM Corp., Armonk, New York, United States). One-way analysis of variance was conducted to assess differences among groups, and Tukey’s test was used for multiple pairwise comparisons. Statistical significance was set at p < 0.05.

## Results

Flexural strength

**Figure 3:**
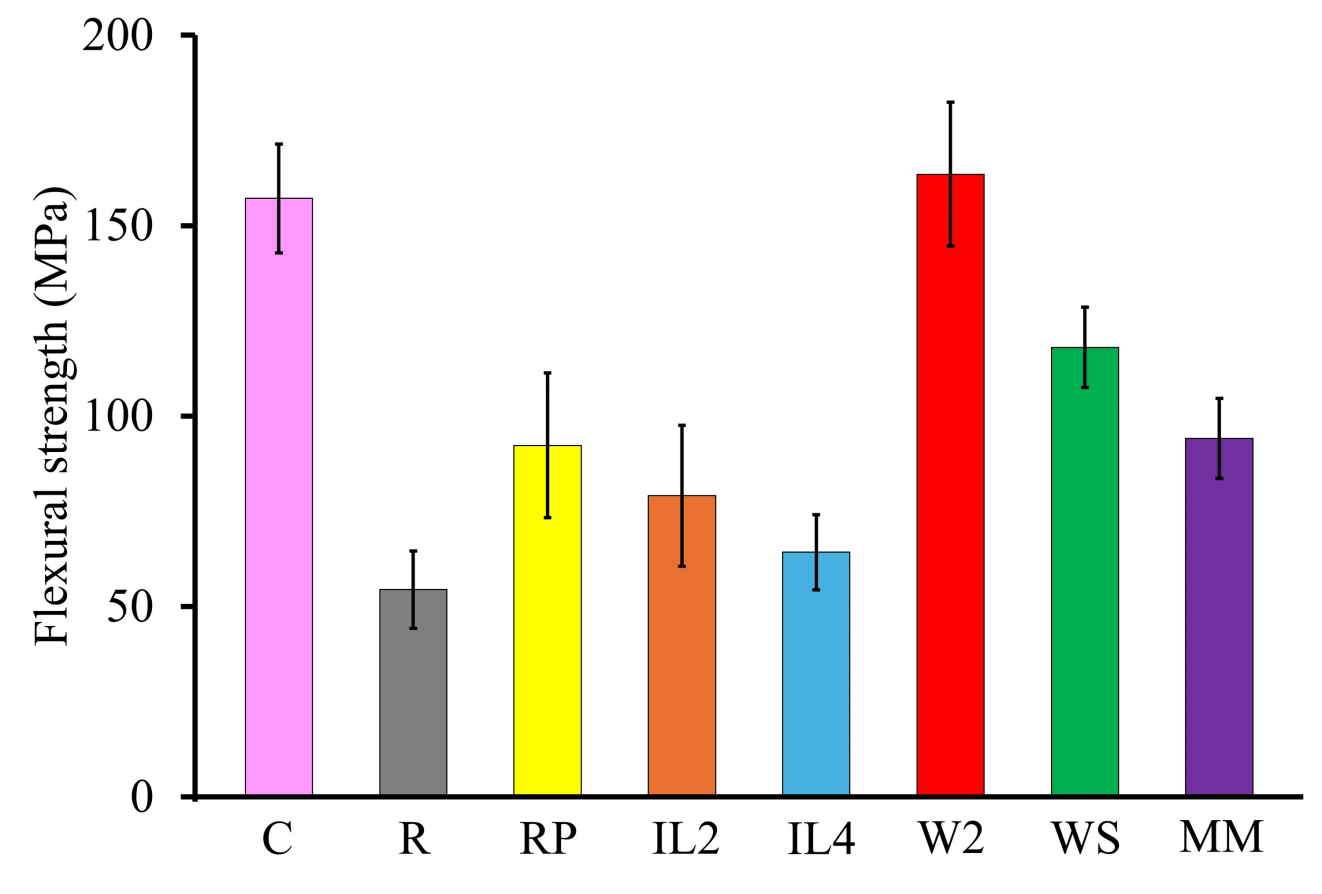
Mean flexural strength of each specimen group

The mean flexural strength of each specimen group is presented in Table 2 and Figure 3, with statistical comparisons shown in Table 3. The C group exhibited a flexural strength of 157.1 ± 14.3 MPa. The lowest value was observed in the R group, which showed 54.4 ± 10.2 MPa, a 65% reduction compared with the C group (p < 0.05). The RP group achieved 92.2 ± 19.0 MPa, representing a 41% decrease relative to the C group (p < 0.05).

**Table 2:** Mean values and relative ratios of flexural strength and maximum deflection with respect to the control group in each specimen group “–” indicates intentionally blank cells.

	Flexural strength (Mpa)	Flexural strength (%)	Maximum deflection (mm)	Maximum deflection (%)
C	157.1 ± 14.3	-	7.7 ± 1.4	-
R	54.4 ± 10.2	-65	2.8 ± 0.3	-64
RP	92.2 ± 19	-41	4.3 ± １	-44
IL2	79.1 ± 18.5	-50	3.1 ± 0.7	-60
IL4	64.3 ± 9.9	-59	2.8 ± 0.4	-64
W2	163.5 ± 18.9	4	4.9 ± 1	-36
WS	118 ± 10.6	-25	3.7 ± 0.5	-52
MM	94.2 ± 10.5	-40	4 ± 0.7	-48

**Table 3:** Results of one-way ANOVA and Tukey’s HSD multiple comparisons for flexural strength among specimen groups The values in the table represent p-values for comparisons with the control group. Statistical analysis was performed using one-way ANOVA (F(7, 120) = 116.211, p < 0.001), followed by Tukey’s post hoc test for multiple comparisons. Statistical significance was set at p < 0.05. “–” indicates intentionally blank cells. HSD: Honestly significant difference

	C	R	RP	IL2	IL4	W2	WS	MM
C	-	-	-	-	-	-	-	-
R	.000	-	-	-	-	-	-	-
RP	.000	.000	-	-	-	-	-	-
IL2	.000	.000	.210	-	-	-	-	-
IL4	.000	.585	.000	.107	-	-	-	-
W2	.931	.000	.000	.000	.000	-	-	-
WS	.000	.000	.000	.000	.000	.000	-	-
MM	.000	.000	1.000	.095	.000	.000	.000	-

Among the IL groups, IL2 (two interlocks) exhibited 79.1 ± 18.5 MPa (50% decrease) and IL4 (four interlocks) showed 64.3 ± 9.9 MPa (59% decrease), both significantly lower than the C group (p < 0.05). The (W2) group was the only group to demonstrate higher mean flexural strength than the C group, with 163.5 ± 18.7 MPa (approximately 4% greater), although this difference was not statistically significant (p > 0.05). The WS and MM groups exhibited 118.0 ± 10.0 MPa (25% decrease) and 94.2 ± 10.5 MPa (40% decrease), respectively, both significantly lower than the C group (p < 0.05).

Maximum deflection

**Figure 4:**
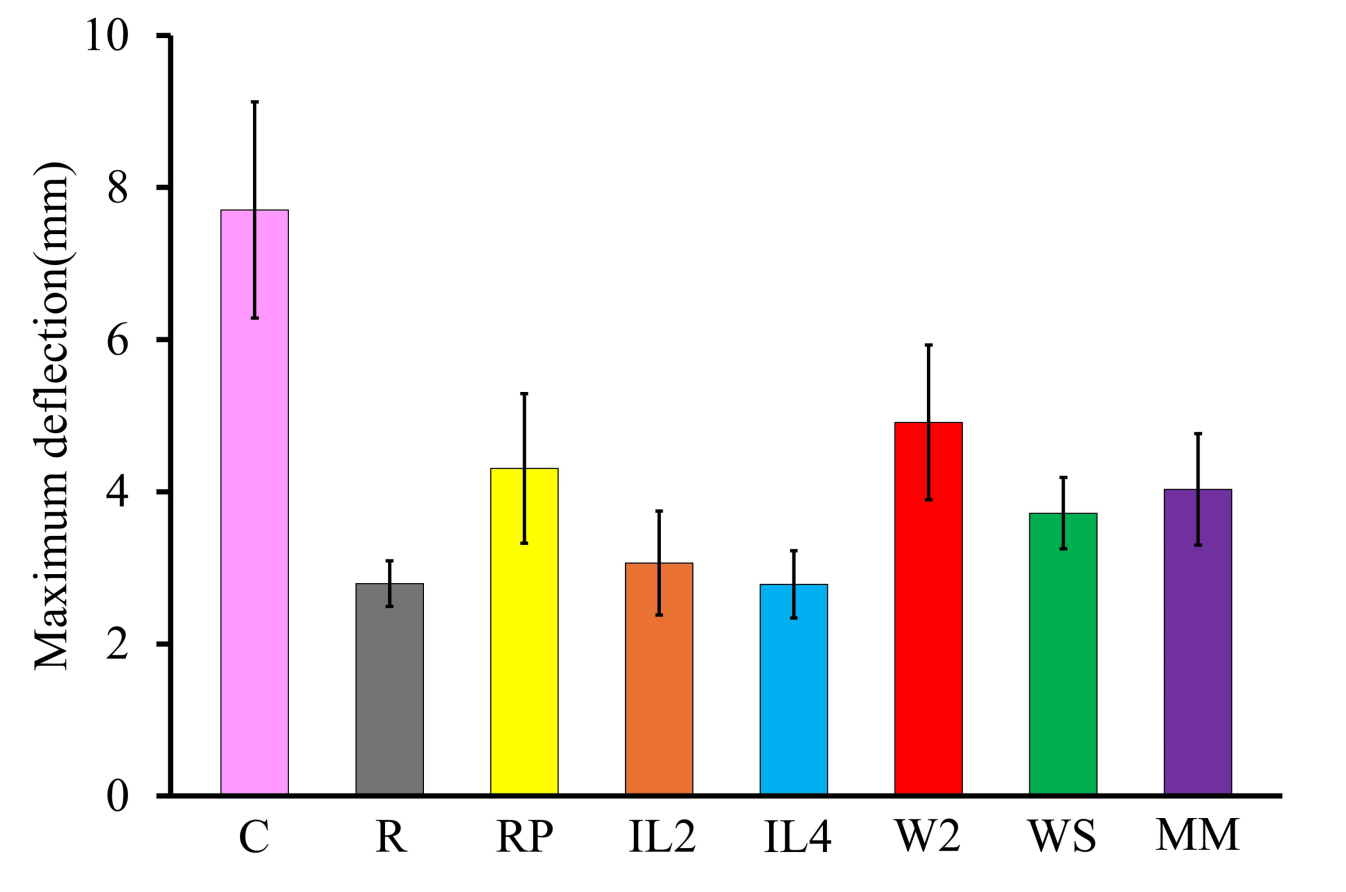
Mean maximum deflection of each specimen group

The mean maximum deflection of each group is summarized in Table 2 and Figure 4, with statistical comparisons shown in Table 4. The C group showed the highest deflection (7.7 ± 1.4 mm). All repair groups demonstrated significantly lower deflection than the C group (p < 0.05). The R group exhibited the smallest deflection (2.8 ± 0.3 mm), representing a 64% reduction. The RP group showed 4.3 ± 1.0 mm (44% decrease), IL2 3.1 ± 0.7 mm (60% decrease), and IL4 2.8 ± 0.4 mm (64% decrease), all significantly lower than the C group (p < 0.05).

**Table 4:** Results of one-way ANOVA and Tukey’s HSD multiple comparisons for maximum deflection among specimen groups The values in the table represent p-values for comparisons with the control group. Statistical analysis was performed using one-way ANOVA (F(7, 120) = 56.833, p < 0.001), followed by Tukey’s post hoc test for multiple comparisons. Statistical significance was set at p < 0.05. “–” indicates intentionally blank cells. HSD: Honestly significant difference

	C	R	RP	IL2	IL4	W2	WS	MM
C	-	-	-	-	-	-	-	-
R	.000	-	-	-	-	-	-	-
RP	.000	.000	-	-	-	-	-	-
IL2	.000	.986	.002	-	-	-	-	-
IL4	.000	1.000	.000		-	-	-	-
W2	.000	.000	.486	.000	.000	-	-	-
WS	.000	.044	.586	.335	.040	.005	-	-
MM	.000	.002	.985	.038	.002	.080	.981	-

The W2 group, which showed flexural strength comparable to the C group, demonstrated 4.9 ± 1.0 mm deflection, representing a 36.4% reduction, the closest to the C group among all repair groups, though still significantly lower (p < 0.05). The WS and MM groups exhibited 3.7 ± 0.5 mm (51.9% decrease) and 4.0 ± 0.7 mm (48.1% decrease), respectively, both significantly lower than the C group (p < 0.05).

## Discussion

In this study, to reflect clinical conditions more accurately, the specimen thickness was set to 2.5 mm, thinner than the ISO standard of 3.3 mm [22]. This thickness corresponds to that of actual denture bases worn by patients and aligns with previous reports on denture repair, thereby enhancing the clinical relevance of the evaluation [19,23]. Under these conditions, the effects of mechanical retention forms and reinforcement materials on the flexural strength and deflection of repaired denture base resin were investigated.

Compared with the C group, the R group exhibited a 65% reduction in flexural strength and a 64% reduction in deflection. This suggests that bonding at the repair interface using autopolymerizing resin alone is insufficient and that stress concentration facilitates early interfacial failure. Previous studies have similarly reported that denture base resin repaired solely with autopolymerizing resin demonstrates lower flexural strength than newly fabricated resin, consistent with the present findings [24,25].

By contrast, the RP group showed a 41% decrease in flexural strength and a 44% decrease in deflection relative to the C group, indicating better preservation of mechanical properties. Primer application likely enhanced adhesion by partially dissolving the resin surface and creating porosity, thereby facilitating monomer interdiffusion from the autopolymerizing resin [25-28]. These results suggest that primer treatment not only improves flexural strength but also preserves post-repair flexibility.

The IL groups (IL2 and IL4) were prepared with mechanical retention features on the fracture surfaces to reinforce the repair. The IL2 group showed significantly higher flexural strength and deflection than the R group, whereas the IL4 group did not demonstrate a significant increase despite the additional grooves. The lower performance of the IL4 group may be attributed to stress concentration at the increased number of grooves, creating additional fracture initiation sites. Previous studies have shown that rough finishing or processing near specimen edges can act as fracture origins and reduce flexural strength, supporting this explanation [9,17,18,28]. Therefore, mechanical interlocking should be applied at moderate intervals near the center of the repair area. Notably, the IL2 group achieved flexural strength comparable to the RP group, suggesting that it could serve as a practical alternative when primer is unavailable, such as in domiciliary care [29].

Regarding reinforcement, the W2 group demonstrated flexural strength comparable to the C group, indicating high reinforcement effectiveness. This group was the only one among the seven repair conditions to surpass the C group, though the difference was not statistically significant. Deflection was also relatively high, with slight opening observed at the fracture site, likely due to sliding of the resin over the parallel wires without surface treatment. Plastic deformation of the wires prevented manual restoration.

The WS group showed a 25% reduction in flexural strength and a 52% reduction in deflection compared with the C group. However, flexural strength remained significantly higher than in the non-reinforced groups, indicating the reinforcing effect of the wire. Unlike the W2 group, fracture opening was minimal, suggesting that the S-shaped configuration provided structural restraint, limiting post-fracture deflection. These observations indicate that wire configuration influences not only strength, but also post-fracture flexibility and stability, highlighting the need to optimize wire placement clinically.

The MM group showed flexural strength similar to the RP and IL4 groups, despite reinforcement. The mesh was placed on the tensile side, and failure was assessed by abrupt load drops during testing, suggesting that recorded fractures may reflect resin failure on the mesh surface. Nevertheless, as with the W2 and WS groups, complete separation of the specimen did not occur, and the thin (0.25 mm) mesh could be manually reshaped after testing. While the mesh’s mechanical reinforcement was limited, it offers clinical advantages: in cases of re-fracture, minimal removal of fractured resin followed by in-situ repair with autopolymerizing resin allows easy re-repair. Its thinness also permits simple trimming with scissors, enhancing its practical applicability.

**Figure 5:**
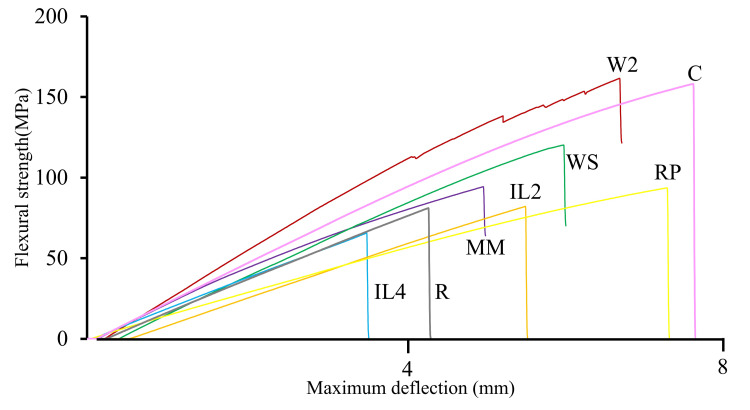
Representative stress–displacement curves of each specimen group

The non-reinforced groups lost almost all flexural strength immediately upon fracture, whereas the reinforced groups retained partial load-bearing capacity (Figure 5). This residual strength allows maintenance of minimal denture function, potentially preserving patient quality of life and facilitating easier subsequent repair.

Overall, once fractured, denture base resin cannot fully regain its original flexural strength after repair, and significant reductions in deflection indicate an inherent risk of re-fracture. Long-term stability of repaired denture base resin therefore requires the combination of primer treatment to enhance adhesion with mechanical retention or reinforcement. Future investigations should explore the use of resin and metal primers and evaluate clinical durability to optimize repair protocols further.

However, this study has several limitations. It was conducted using standardized test specimens under controlled laboratory conditions, which cannot fully replicate the complex intraoral environment or the patient-specific variables encountered in clinical practice. Furthermore, the evaluation was limited to measurements of flexural strength and deflection using a three-point bending test and did not include fatigue testing, cyclic loading, or thermal cycling to simulate intraoral conditions. In addition, although heat-cured resin is commonly used for denture fabrication, both the test specimens and the repaired areas in this study were made using auto-polymerizing resin. The use of a specific primer may also limit the generalizability of the findings, as different primer formulations could affect adhesion and mechanical performance; future studies incorporating surface analysis would be valuable to confirm these mechanisms. Operator variability in groove or wire placement was not quantified, which may affect the reproducibility of the repair procedure. Future studies should include cyclic loading experiments and clinical follow-up evaluations to verify the long-term effectiveness and clinical applicability of the proposed repair methods. Moreover, to make the findings more clinically relevant, it will also be necessary to conduct comparative studies using test specimens fabricated with both heat-cured and auto-polymerizing resins and to evaluate the effects of different primer formulations.

## Conclusions

The results of this study indicated that primer treatment, appropriate mechanical interlocking geometry, and reinforcement materials can significantly improve the mechanical properties, specifically flexural strength and maximum deflection, of repaired denture base resin. Although these methods are already employed in chairside or home-visit repairs, the observed reduction in flexibility after repair indicates a potential risk of re-fracture. Further investigations involving resin and metal primers, fatigue testing, and long-term clinical evaluation are necessary to establish reliable and durable clinical repair protocols.
